# Development of an Emergency Department–Based Intervention to Expand Access to Medications for Opioid Use Disorder in a Medicaid Nonexpansion Setting: Protocol for Engagement and Community Collaboration

**DOI:** 10.2196/18734

**Published:** 2021-04-29

**Authors:** Lauren A Walter, Li Li, Joel B Rodgers, Jennifer J Hess, Rachel M Skains, Matthew C Delaney, James Booth, Erik P Hess

**Affiliations:** 1 Department of Emergency Medicine University of Alabama at Birmingham Birmingham, AL United States; 2 Department of Psychiatry and Behavioral Neurobiology University of Alabama at Birmingham Birmingham, AL United States; 3 Department of Emergency Medicine Vanderbilt University Nashville, TN United States

**Keywords:** opioid use disorder, mediation for opioid use disorder, emergency medicine, buprenorphine, peer support services

## Abstract

**Background:**

The opioid epidemic has disproportionately impacted areas in the Appalachian region of the United States. Characterized by persistent Medicaid nonexpansion, higher poverty rates, and health care access challenges, populations residing in these areas of the United States have experienced higher opioid overdose death rates than those in other parts of the country. Jefferson County, Alabama, located in Southern Appalachia, has been especially affected, with overdose rates over 2 times greater than the statewide average (48.8 vs 19.9 overdoses per 10,000 persons). Emergency departments (EDs) have been recognized as a major health care source for persons with opioid use disorder (OUD). A program to initiate medications for OUD in the ED has been shown to be effective in treatment retention. Likewise, continued patient engagement in a recovery or treatment program after ED discharge has been shown to be efficient for long-term treatment success.

**Objective:**

This protocol outlines a framework for ED-initiated medications for OUD in a resource-limited region of the United States; the study will be made possible through community partnerships with referral resources for definitive OUD care.

**Methods:**

When a patient presents to the ED with symptoms of opioid withdrawal, nonfatal opioid overdose, or requesting opioid detoxification, clinicians will consider the diagnosis of OUD using the Diagnostic and Statistical Manual of Mental Disorders (fifth edition) criteria. All patients meeting the diagnostic criteria for moderate to severe OUD will be further engaged and assessed for study eligibility. Recruited subjects will be evaluated for signs and symptoms of withdrawal, treated with buprenorphine-naloxone as appropriate, and given a prescription for take-home induction along with an intranasal naloxone kit. At the time of ED discharge, a peer navigator from a local substance use coordinating center will be engaged to facilitate patient referral to a regional substance abuse coordinating center for longitudinal addiction treatment.

**Results:**

This project is currently ongoing; it received funding in February 2019 and was approved by the institutional review board of the University of Alabama at Birmingham in June 2019. Data collection began on July 7, 2019, with a projected end date in February 2022. In total, 79 subjects have been enrolled to date. Results will be published in the summer of 2022.

**Conclusions:**

ED recognition of OUD accompanied by buprenorphine-naloxone induction and referral for subsequent long-term treatment engagement have been shown to be components of an effective strategy for addressing the ongoing opioid crisis. Establishing community and local partnerships, particularly in resource-limited areas, is crucial for the continuity of addiction care and rehabilitation outcomes.

**International Registered Report Identifier (IRRID):**

DERR1-10.2196/18734

## Introduction

### Background

The Appalachian region of the Eastern United States has been disproportionately affected by the opioid epidemic. In 2017, the death rate for opioid overdoses in Appalachian counties was 72% higher than that in non-Appalachian counties [1]. The number of opioid-related deaths in Appalachia has now surpassed the number of deaths from motor vehicle accidents [2]. A Medicaid nonexpansion state, Alabama, is located in Southern Appalachia and has been particularly affected by the opioid epidemic. Since 2014, Alabama has led the nation with the highest rate of opioid prescriptions (107.2 prescriptions for every 100 persons), nearly two-fold greater than the national average [3]. Following efforts implemented by the US Drug Enforcement Administration (DEA) in 2010 to decrease opioid prescription rates, the demand for more potent opioids such as heroin and fentanyl increased, resulting in an unintended increase in the number of overdose deaths nationally [4]. This trend also occurred in Alabama, where there was a significant (11.1%) increase in the age-adjusted rate of drug overdose deaths from 2016 to 2017 [3]. Jefferson County in Alabama is a hot spot for opioid use disorder (OUD) and its complications. An analysis of naloxone administration in 2016 revealed that Jefferson County had 48.8 overdoses per 10,000 persons, a rate significantly higher than the statewide average of 19.9 per 10,000 persons [5]. In 2018, the Jefferson County Coroner’s office confirmed 228 illicit drug deaths, 171 of which were because of an opioid overdose [6].

Patients with OUD often present to emergency departments (EDs) to treat opioid-related conditions, including nonfatal opioid overdose [7]. Persons with OUD are high users of ED services. People who inject drugs have been shown to use EDs over 3 times more frequently than the general population [8]. Mortality after ED discharge following nonfatal opioid overdose has been shown to be as high as 5.5% within 1 year, highlighting a vulnerable population that may benefit from ED intervention [9]. Beyond acute medical stabilization, ED interventions for this patient population have traditionally been limited to brief cessation counseling and referral for drug rehabilitation. Recently, a brief negotiation interview (BNI), the treatment of withdrawal with buprenorphine-naloxone as appropriate, and referral to treatment have been shown to be an effective strategy for OUD treatment. In a randomized trial comparing referral alone with BNI plus referral and BNI plus referral plus buprenorphine, ED patients randomized to the BNI plus referral plus buprenorphine arm had a nearly two-fold higher rate of retention in addiction treatment and significantly lower use of inpatient addiction services at 30 days [10]. Despite this evidence, the widespread adoption has lagged. The implementation gap is particularly marked in Alabama, where, according to the Substance Abuse and Mental Health Services Administration (SAMHSA) database and other publicly available data, only 2.18% (138/6342) of physicians had completed the Drug Addiction Treatment Act (DATA) 2000 waiver training, a requirement to prescribe medications for opioid use disorder (MOUD) such as buprenorphine [11,12]. Data elsewhere suggest that an even smaller percentage of X-waivered clinicians actively use their X-waiver to provide MOUD [13]. The shortage of clinicians providing MOUD in Alabama is profound.

Similarly, although the ED has long been recognized as the health care system’s “front door,” providing critical access and referral to primary care and specialty services, it often does not translate to effective linkage to substance use disorder (SUD) treatment. In Appalachia, an insufficient supply of behavioral and public health services targeting opioid misuse contributes to higher rates of opioid misuse and mortality in the region [2]. Engagement in long-term rehabilitation and MOUD, however, are an integral part of the recovery process for patients with OUD. Patients with SUD who are directly admitted to a treatment facility have been found to be 30 times more likely to enroll than those who were indirectly referred, as is most often the case in the ED setting [14]. An additional important consideration is the resource-limited setting in which many EDs in non-Medicaid expansion states operate, particularly when considering treatment options for low-income, uninsured patients. Therefore, developing community partnerships that facilitate linkage to care is a critical component of OUD treatment after discharge from the ED.

### Objective

The overall objective of this implementation project is to increase the number of persons with OUD who present to either of 2 academic EDs in Jefferson County to receive MOUD and community treatment referral, thereby improving OUD outcomes in this region. We will accomplish this objective by initiating buprenorphine-naloxone in the ED, activating bedside referral services, and linking patients to longitudinal addiction care. Table 1 outlines the goals and objectives of this SAMHSA-funded public health intervention (SAMHSA Award #1H79TI081609). As the first ED-initiated medication-assisted treatment (MAT) program in Alabama, a resource-limited, non-Medicaid expansion state, we aim to demonstrate the feasibility and effectiveness of an ED-MOUD program in this region and identify barriers and potential solutions to expand access to MOUD further. We hope our experience will inform other resource-limited regions in the United States that may be seeking to improve access to MOUD and treatment options for persons with OUD.

**Table 1:** Goals and objectives for the Emergency Department-Medication for Opioid Use Disorder Therapy Demonstration Project in Jefferson County, Alabama.

Goals for Jefferson County, Alabama	Measurable objectives
Increase the number of clinicians with DATA^a^ 2000 training to identify and treat patients with OUD^b^	Obtain DATA 2000 waiver training in at least 75% of ED^c^ attending and licensed resident physicians by February 28, 2020. (25 UAB-ED^d^ MDs^e^ by July 2, 2019; an additional 30 MDs by February 28, 2020).
Develop and implement an electronic evidence-based induction and referral to treatment protocol initiated in the ED	Implement an electronic evidence-based order set to standardize OUD diagnosis, determine the need for buprenorphine induction, activate bedside referral services, and initiate outpatient MOUD^f^ in the University and Highlands EDs by July 2, 2019.
Operationalize the protocol to increase the number of individuals with OUD referred for MOUD	Treat and refer 272 individuals with OUD for MOUD over the 3-year project period (56 in year 1+108 in year 2+108 in year 3).
Improve retention in care for individuals who have been diagnosed with OUD	Increase treatment completion rates by individuals with OUD throughout the program by 5%-10% each year (year 1=baseline).
Decrease opioid overdose–related deaths	In collaboration with other community-based initiatives and public health interventions, decrease opioid overdose–related deaths in Jefferson County by 30% over 3 years.

^a^DATA: Drug Addiction Treatment Act.

^b^OUD: opioid use disorder.

^c^ED: emergency department.

^d^UAB-ED: University of Alabama at Birmingham Hospital Emergency Department.

^e^MD: Medicine Doctor.

^f^MOUD: medications for opioid use disorder.

## Methods

### Study Population

The population for this project includes patients seeking treatment for OUD who present to either of 2 University of Alabama at Birmingham (UAB) Hospital EDs located in Jefferson County. The University Hospital ED is a tertiary care academic ED with >70,000 annual patient visits in 2018, and the Highlands Hospital ED is an urban community ED with 30,000 annual patient visits in the same year. To estimate the number of persons potentially impacted by this public health intervention, we conducted an *International Classification of Diseases*, *Tenth Revision*, search of our electronic health record (EHR) from January 1, 2018, to December 31, 2018 [15]. We identified 2395 unique patients who presented to the ED to treat nonfatal opioid overdose, opioid withdrawal, or opioid detoxification. This prevalence rate, approximately 240 per 10,000 people, is above the national average of opioid-related ED visits [16]. The mean age was 39.8 years (SD 12.4 years), and most patients were male (1432/2395, 59.79%) and White (1861/2395, 77.70%); 50.02% (1198/2395) of the patients were uninsured (Table 2).

**Table 2:** Baseline characteristics of emergency department patients seen for opioid overdose, opioid withdrawal, or seeking detoxification in 2018 (n=2395).

Characteristic		Value
Age, mean (SD)		39.8 (12.4)
Male sex, n (%)		1433 (59.83)
**Race, n (%)**		
	White or Caucasian	1860 (77.66)
	Black or African American	485 (20.25)
	Other	50 (2.09)
**Insurance status, n (%)**		
	Privately insured	380 (15.87)
	Publicly insured (Medicare or Medicaid)	817 (34.11)
	Self-pay or uninsured	1198 (50.02)

The geographic catchment area for this initiative, Jefferson County, is the largest and most populous county in the state of Alabama, encompassing the city of Birmingham and 29 additional municipalities. Jefferson County has a total area of 1124 square miles and a population of 659,300, of which 44% are African American, 53% Caucasian, and 17% live below the federal poverty level [17].

Admitted patients will be excluded from this study. Variability in inpatient treatment plans and logistical inability to consistently provide a *warm handoff* to a community partner (see *Community Partnerships* section below) make enrollment of admitted patients less feasible in our setting. Other vulnerable populations, including prisoners, minors, pregnant patients, or those unable to consent, will also be excluded.

### Implementation Plan

Enrollment will occur in the ED 24 hours per day, 7 days per week. To be feasible in our context, the protocol developed by D’Onofrio et al [10] will be modified in the following ways: first, given the large number of patients who present to UAB EDs with a primary complaint of nonfatal opioid overdose, opioid withdrawal, or requesting opioid detoxification, these chief complaints will serve to identify patients with suspected OUD rather than a universal screening method. Emergency physicians’ primary role in this project is to identify patients with OUD who may be eligible for ED-initiated MOUD and referral to treatment. If a patient meets 4 or more *Diagnostic and Statistical Manual of Mental Disorders* (Fifth Edition) (*DSM-5*), criteria (moderate to severe OUD), physicians will be asked to conduct a BNI to explore the individual’s motivation to engage in treatment (see the *Physician Education and Engagement* section; DATA 2000 waiver training plus supplemental physician education encompasses *DSM-5* OUD criteria and BNI training). The BNI typically takes ≤5 to 15 minutes [18]. If the patient appears motivated, the physician will activate an order set within the EHR to notify the research staff in real time. Research staff will assess the patient for eligibility, conduct enrollment, and assist with linkage to care via direct communication with community referral partners. Physicians may provide a dose of buprenorphine-naloxone in the ED if the patient is in active opioid withdrawal (may be repeated if necessary for ongoing withdrawal symptoms) and will be asked to provide a buprenorphine-naloxone *bridge* prescription at the time of patient discharge from the ED [10]. Patients provided with a buprenorphine-naloxone prescription will also be provided with in-person (from the provider) and handout instructions regarding buprenorphine induction and titration. Patients who are not eligible or who do not wish to enroll will be provided the appropriate existing standard of care, including a list of referral resources for OUD for self-access at the time of ED discharge.

It must be noted that the prescription drug monitoring program (PDMP), a statewide controlled substance prescription database, is seamlessly integrated into the EHR at UAB and is now the standard of care in our hospitals. The PDMP for each patient is visible to all providers registered with the Alabama PDMP without the need to access an external website. A review of PDMP records before initiation of MOUD is an approach recommended by a recent American College of Emergency Physicians policy statement [19]. The PDMP will be consistently used for this study to (1) assist providers in the diagnosis of OUD, when applicable (eg, multiple overlapping opioid prescriptions from multiple providers), and (2) thoughtfully consider the treatment plan of patients who may return to the ED for MOUD refill prescriptions (see the *Plans to Mitigate Risk of Diversion* section).

### Clinical Protocol

To alert research staff to potential study participants, we developed a custom OUD order set in Cerner that delivers automated, real-time, electronic notifications to research personnel when activated by an emergency physician (Table 3). A departmental protocol guides the initiation of the OUD order set by physicians at any time a patient meets the *DSM-5* criteria for at least moderate OUD. Following the electronic alert, trained research staff, consisting of a research coordinator and/or research assistant, collaborate directly with the ordering the clinician and the patient to ascertain appropriateness for study inclusion. Research staff will assess patients for enrollment eligibility, complete the enrollment process, and facilitate handoff to community referral partners. If enrolled, research staff will also contact peer navigators to be dispatched to the bedside by the Recovery Resource Center (RRC)—a Jefferson County community substance abuse treatment coordinating center—to facilitate linkage to outpatient addiction treatment.

**Table 3:** Custom opioid use disorder electronic health record order set.

UED^a^ opiate use disorder (initiated pending)					Order
**Patient care**					
	Communication order nursing			Complete COWS^b^ assessment	
	Communication order nursing			Verify the patient’s contact information for follow-up purposes	
**Medications**					
	**COWS 8-13**				
		Buprenorphine-naloxone (buprenorphine [dosed with naloxone])	4 mg, Tab-SL^c^, sublingual, every 1 hour; now, PRN^d^, other (see comment below regarding observation prior to second dose), 2 doses Administer 4 mg now. Observe patient for 45-60 min. If no adverse events, administer second dose		
	**COWS >13**				
		Buprenorphine-naloxone (buprenorphine [dosed with naloxone])	8 mg, Tab-SL, sublingual, once, now 0.5 mg naloxone per each 2 mg buprenorphine		
**Discharge prescriptions**					
	Buprenorphine-naloxone (buprenorphine-naloxone 8 mg-2 mg sublingual tablet)			=1 tablet, sublingual, BID^e^, #14 tablets, refill 0	
	Naloxone intranasal (take-home supply)				
	Buprenorphine-naloxone (buprenorphine-naloxone 8 mg-2 mg sublingual tablet)			=1 tablet, sublingual, BID, #20 tablets, refill 0	
**Laboratory**					
	Drugs of abuse profile (urine drug screen)			Urine	
	Comprehensive metabolic panel			Blood	
	Order urine pregnancy test for females aged 15-55 years			Urine	
**Consults**					
	Consult to social services			Other (use special instructions); evaluate opioid use disorder, treatment, and referral in the emergency department	

^a^UED: university emergency department.

^b^COWS: Clinical Opioid Withdrawal Scale.

^c^Tab-SL: tablet-sublingual.

^d^PRN: pro re nata (as needed).

^e^BID: bis in die (twice daily).

Concomitantly, prompted by a nursing order in the OUD order set, the nursing staff will assess the presence and severity of opioid withdrawal symptoms using the Clinical Opioid Withdrawal Scale (COWS) [20]. If the COWS score is between 8 and 13 (moderate withdrawal), a 4 mg-1 mg sublingual dose of buprenorphine-naloxone will be offered to the patient for induction. The patient will be observed for 1 hour, and a second 4 mg/1 mg dose will be provided if needed based on symptomatic response and repeated COWS score assessment. If the COWS score is >13 (severe withdrawal), an 8 mg-2 mg sublingual dose of buprenorphine-naloxone will be offered for induction, and the patient will be observed for symptomatic improvement [21]. Buprenorphine-naloxone administered in the ED will be stored in Omnicell automated medication-dispensing cabinets located in the ED for efficient access and delivery to patients in withdrawal. If the patient is agreeable to engaging in outpatient addiction treatment, a 10-day prescription for buprenorphine-naloxone, 8 mg-2 mg sublingual BID (twice daily), will be offered for take-home induction bridge to outpatient MOUD (10 days has been determined to be within the current average time to follow-up for local MOUD clinics). In addition, a take-home intranasal naloxone kit will be provided along with instructions on use and general overdose education (Multimedia Appendix 1). The buprenorphine-naloxone prescription will be filled on site by the hospital pharmacy, allowing the patient to be discharged with the medication in hand. The take-home naloxone kit and supply of buprenorphine-naloxone will also be provided at no cost to enrolled patients. The study cost of buprenorphine-naloxone is US $6 per pill. The intranasal naloxone kits are provided free of charge to our department by the Jefferson County Health Department.

### Medical Clearance

Before buprenorphine-naloxone induction and activation of bedside referral services, patients will undergo routine medical screening and clearance consisting of a comprehensive metabolic panel, urine drug test (UDT), and a urine pregnancy test in female patients with childbearing potential. Although the only true contraindication to buprenorphine is a hypersensitivity reaction to the medication, dose modification in the setting of hepatic dysfunction will be considered. Urine drug screening will allow clinicians to detect patients who are malingering and/or seeking to divert buprenorphine-naloxone. Pregnant patients with OUD in active withdrawal or seeking addiction care receive a consultation for psychiatric addiction services in the ED. Preexisting institution-specific services offer focused monitoring and intervention for this specific population to minimize the risk of neonatal abstinence syndrome and miscarriage and optimize pregnancy outcomes [22]. As such, pregnant patients will be excluded from this study.

### Plans to Mitigate the Risk of Diversion

Only buprenorphine-naloxone, which has a lower risk of diversion than methadone, will be administered by ED staff per DATA 2000 waiver training regulations [23]. The Alabama PDMP will be checked before each buprenorphine-naloxone prescription (as described above). Each prescription’s amount and duration will be documented in the EHR and communicated to the RRC and subsequently to the receiving Alabama Department of Mental Health (ADMH) certified treatment facility. Physician engagement and education (see *Physician Education and Engagement* section) include standard DATA 2000 waiver training, which encompasses UDT interpretation. Therefore, the UDT collection will be included in the OUD EHR order set, and result consideration will be advised per departmental protocol before administering or prescribing buprenorphine-naloxone. Likewise, consideration of the PDMP will be strongly encouraged for any provider prescribing buprenorphine-naloxone. No specific restrictions or guidelines will be provided during this study concerning either the PDMP or UDT analysis beyond the standard DATA 2000 training. Rather, they are additional resources available to our physicians for both OUD diagnosis and evaluation of suspected diversion.

ADMH-certified facilities that receive OUD patients are required to independently develop, maintain, and implement diversion control plans. We will also limit the duration of the buprenorphine-naloxone prescription to 10 days, with an option for a subsequent *as needed* 7-day refill to balance 2 potential factors inherent to local substance abuse treatment referral: (1) waiting time before outpatient addiction services are available and (2) the risk of diversion associated with prescriptions of prolonged duration.

Despite linkage support and efforts, there may be occasions when a patient fails to link to outpatient MOUD. In these circumstances, the patient may return to the ED seeking reengagement in the program and a MOUD refill. In these instances, research staff engagement can be used to identify and potentially overcome specific barriers to the initial treatment plan. Recidivism will also be monitored. However, the decision to provide a return patient with a refill prescription will be determined on an individual basis and at the discretion of the prescribing physician.

### Physician Education and Engagement

In the state of Alabama, 2 hours of opioid-specific continuing medical education are required every 2 years, which aligns with the goal of UAB-attending physicians to obtain DATA 2000 waiver training. In the months leading up to the project start date, the principal investigator provided brief presentations at monthly emergency medicine faculty meetings to describe the evidence supporting MOUD initiation in the ED—the high incidence of opioid overdose and death in Jefferson County—and advocate for the importance of a concerted initiative centered around the needs of patients with OUD. In addition, coauthors (LAW and JJH) developed 4 hours of didactic material to deliver to emergency medicine residents in a single, half-day format, including presentations, small group case discussions, simulations, and a presentation by leadership from the RRC. In an effort to encourage learner engagement and provide a general overview and introduction of the in-person didactic, we created and released a peer-reviewed podcast that is available on iTunes and Soundcloud [24,25]. Finally, as a public health intervention consistent with the mission and values of the UAB Department of Emergency Medicine, the Emergency Medicine Department Chair mandated the faculty completion of X-waiver training.

### Community Partnerships

The RRC is a local addiction coordinating center that facilitates intake assessment and substance abuse referral and placement for all-comers, regardless of insurance status. Following patient intake and assessment, the RRC uses local resources and networks to direct the patient to the most appropriate definitive MOUD continuing care option available. The engagement of the RRC as a local clinical referral partner in this project was a crucial initial step. Assisting individuals with substance abuse in navigating the treatment system is an RRC mission. Formalizing a referral process with the RRC as a part of this project fulfilled a critical need for our patients and helped them fulfill their vision as a valuable community resource. As described above, when an OUD patient is identified by ED staff, the RRC will be engaged by research staff to dispatch a trained peer recovery specialist to ensure a *warm handoff* between ED and RRC team members and initiate referral procedures for follow-up. A warm handoff is a direct transfer of care between 2 members of a health care team that typically occurs in front of the patient. This seamless transition in care has been shown to decrease the time to enrollment in recovery programs and improve recovery prospects [26]. Following study enrollment and contact by research staff, the peer recovery specialists meet directly with the patient in the ED, creating a direct personal link to the RRC for subsequent linkage to care steps. Peer recovery specialists or *peer navigators* are individuals in sustained recovery from OUD who have undergone a certification process involving 40 hours of specialized substance abuse training. Peer navigators are funded by the RRC, which is supported by the local county health department and the Birmingham Crisis Center. The peer navigators provide direct assistance to the patients to facilitate access and intake at the RRC (eg, physically accompanying them on the same day to RRC intake or sharing contact information to use next-day contact to ensure RRC follow-up). RRC staff will perform a detailed intake assessment, including the American Society for Addiction Medicine and Government Performance and Results Modernization Act (GPRA) assessments, and initiate a referral to an ADMH-certified treatment facility for the continuation of MOUD. ADMH-certified treatment facilities deliver comprehensive psychosocial services, including drug counseling, recovery support services, and behavioral therapies, in addition to medication. Within our local community, we currently have 4 inpatient facilities that provide opioid detoxification and rehabilitation services, including MOUD, comprising approximately 200-plus inpatient beds with varying wait times for intake (immediate to 1 week). In addition, we have 5 outpatient facilities, several of which overlap with the aforementioned inpatient facilities, with additional capacity for weekly (during new induction) and monthly (maintenance) MOUD services. Wait times for outpatient linkage averages 3 days to 2 weeks, depending on the facility.

In addition, per RRC standard practice, peer navigators will continue to facilitate the patient’s care until the patient has obtained MOUD clinic follow-up or the patient no longer wishes to be contacted by the peer navigator or is otherwise unreachable.

Letters of commitment from referral partners (see the *Data Collection and Evaluation* section) allowed communication between research staff, the RRC, and MOUD referral partners to be bidirectional. In addition, the UAB research faculty participate in the RRC oversight committee, which regularly engages community substance abuse treatment partners in the discussion and monitoring of local MOUD resources.

### Data Collection and Evaluation

The GPRA of 1993, which was revised in 2010, requires SAMHSA grantees to collect and report performance data using standardized measurement tools [27]. GPRA tools gather extensive patient-specific information, including basic demographics, substance use and abuse, mental health and physical health functioning, and other key variables. The baseline GPRA data collection will be coordinated and completed by the dedicated research staff during the initial ED visit. In-person follow-up data collection interviews at 3 and 6 months postintake and at discharge from addiction treatment will be collected by a contracted third-party vendor, Community Tracking Services (CTS). Follow-up data (eg, current address and contact information) will be obtained through data-sharing follow-up efforts led by referral facilities, including the RRC. Discharge from addiction treatment as an event is technically determined by each respective MOUD clinic and will align with individual patient treatment plans. However, for this project’s purposes, it will be defined as when the patient is no longer maintained on MOUD maintenance therapy, including concomitant psychosocial support services, and therefore no longer an active patient at the MOUD clinic.

Contact barriers for obtaining follow-up data have been considered. Research and CTS staff will rely heavily on MOUD support partners and referral centers, namely, the RRC and the RRC peer navigators, to ensure accurate and up-to-date contact information for enrolled patients is maintained. In addition, contact information for family and friends will also be obtained with patient consent at the time of enrollment. MOUD clinic referral partners will also collect additional contact information.

Project data will be stored in a secure REDCap (Research Electronic Data Capture) database housed on UAB-encrypted servers [28]. Per requirements, project data are also entered and stored in SAMHSA’s Performance Accountability and Reporting System, a web-based data entry, reporting, and technical assistance portal. Formal institutional review board (IRB) protocols and data use agreements have been executed between UAB and non-UAB partners to ensure confidentiality, human subject protection, and Health Insurance Portability and Accountability Act compliance. Written consent will be obtained according to federal confidentiality law and regulation 42 Code of Federal Regulations, Part 2, to exchange health information between UAB, the RRC, and ADMH-certified MOUD treatment facilities to coordinate care delivery. Program evaluation activities will measure (1) the fidelity of program delivery to the timeline, (2) delivery of DATA 2000 training as targeted, (3) data demonstrating implementation of evidence-based practices (eg, MOUD and peer support services), and (4) GPRA data collection. MOUD referral partners and CTS are permitted to collect and share data under the provision of a signed letter of commitment, which describes the respective data collection and sharing roles and responsibilities. Letters of commitment were included in the IRB protocol.

The outcomes and measurable project goals are listed in Table 1. Progress toward the first goal will be evaluated by comparing the percentage of documented DATA 2000 waivers with the census of ED physicians with current DEA certificates. Achievement of goal 2 will be assessed by electronically tracking when the protocols are used and the EHR review. Goal 3 will be evaluated based on GPRA interview data collected at the time of referral to the RRC. Goals 4 and 5 will be evaluated by comparing 3-month, 6-month, and discharge follow-up information from year to year. Goal 5 will also be evaluated by comparing opioid-related deaths at the end of years 2 and 3 from Jefferson County Coroner reports, with the average between 2014 and 2017 baseline years.

### Data Analysis and Management

Analysis of outcome data will include descriptive statistics, including tables that summarize quantitative data and the number of clients who complete treatment compared with those who do not. We will test for differences in a variety of demographic and socioeconomic variables, including racial and ethnic groups. Client data will be analyzed at the time of enrollment, 3 and 6 months post enrollment, and at the time of discharge from local substance abuse treatment facilities. Program outcomes will be monitored quarterly. Qualitative data will be analyzed according to the project evaluator’s procedures, as appropriate for the variable collected.

## Results

Funded in February 2019, this study is in the active enrollment and data collection phase. The UAB IRB approved it in June 2019, and data collection and enrollment began on July 7, 2019. The project end date is projected to be February 26, 2022. As of February 2019, we have enrolled 79 participants. We anticipate the results of the public health intervention to be published in the summer of 2022.

## Discussion

### Overview

Across the United States, the opioid epidemic continues to rage, disproportionately affecting particular areas of the nation, including Appalachia. Appalachia is characterized by a high poverty rate (16.3% as compared with 14.6% nationally) and limited access to subspecialty care (42% of the Appalachian population is rural compared with 20% of the national population), and Appalachian counties have more health care costs coupled with coverage and access disparities than the rest of the United States [29]. Alabama is a non-Medicaid expansion state, which further exacerbates these health care disparities [30].

These characteristics highlight the potential impact of an ED-based intervention for patients with OUD at UAB, the largest and only tertiary care academic medical center in the state of Alabama. They also highlight the necessity of collaboration and community partnerships for a public health intervention such as this to be successful. Many individuals who require addiction care comprise an underserved population with limited access to health care. Our local community partner, the RRC, coordinates care for over 1000 patients with OUD or SUD annually and refers their clients for MOUD, demonstrating an efficient model of care. The linkage of this community resource directly to OUD patients in the ED is critical to ensure continuity of care. EDs have previously served as effective venues to raise awareness of and linkage to care for similar stigmatizing and deadly conditions—specifically HIV and hepatitis C virus [31,32]. The successful implementation of the public health intervention described herein further demonstrates that the ED setting represents a unique opportunity to engage with the community and improve the health of the public [33].

### Future Directions

As we implement the ED-MOUD protocol, a greater number of individuals with OUD will be identified and referred to treatment, increasing the demand for MOUD. As there is a shortage of addiction providers in our region, there is an urgent need to increase the number of clinicians with DATA 2000 training who can effectively provide MAT longitudinally in collaboration with UAB addiction specialists.

An integrated hub and spoke opioid treatment network was developed in Vermont, which may be appropriate for modification and application in our region [34]. In the Vermont model, hub clinics with addiction medicine expertise assess patients’ medical and psychiatric needs at intake, induce patients with buprenorphine, and determine the most appropriate treatment placement. Once patients are stable on buprenorphine, they are referred to a spoke provider. Spokes have direct access to hubs for consultation, screening, and MOUD delivery, and transfers between spokes and hubs can be bidirectional. Any waivered physician can become a spoke provider, hoping that with the extra support of the hub, the MOUD capacity will increase. In Alabama, a non-Medicaid expansion state, we will need to modify the Vermont spoke and hub model to be financially sustainable. In Jefferson County, Federally Qualified Health Centers (FQHCs), community-based health care providers that receive funds from the Health Resources and Services Administration Health Center program, provide primary care services in underserved areas and have the potential to act as spokes in a modified Vermont model [35].

A recent additional grant from SAMHSA will enable the investigative team to establish a Provider Clinical Support System Data 2000 waiver training program at our institution along with a program to train primary care clinicians to deliver MOUD to patients with OUD. This program will facilitate collaboration with FQHCs in Jefferson County to provide addiction training, oversight, and consultation to expand primary care providers’ scope of practice to include MOUD. In addition, we are developing a telemedicine-assisted consultation model to support primary care providers as they deliver care to individual patients. We hope that these additional training programs and models will expand MOUD capacity in Alabama, similar to the Vermont model.

We are also exploring opening a MOUD bridge clinic at UAB to manage patients between the time of identification in the ED and linkage to longitudinal addiction treatment. Such a clinic would also provide an opportunity to incorporate multidisciplinary engagement, including medical social workers and case management services, strategically identifying and addressing patients’ social determinants of health to facilitate treatment of OUD. It will also provide a supervised venue for training physicians in the complexities of MOUD, equipping them to continue these addiction practices as they are subsequently hired throughout the state in rural communities.

### Conclusions

Traditionally a niche reserved for psychiatrists or addiction medicine specialists, treatment of OUD with MOUD is expanding to emergency clinicians, primary care clinicians, and other health care providers. EDs are the tip of the spear where societal problems meet health care, and the intersection between the opioid epidemic and the emergency care system highlights this reality. Initiating MOUD in the ED is an evidence-based approach for improving health outcomes in OUD. Community collaboration is critical for developing a feasible, cost-effective, and sustainable approach to combat the opioid epidemic in resource-limited regions.

## Appendix

Multimedia Appendix 1 Emergency departments’ medications for opioid use disorder induction flowsheet.

## Abbreviations

ADMH: Alabama Department of Mental Health
BNI: brief negotiation interview
COWS: Clinical Opioid Withdrawal Scale
CTS: Community Tracking Services
DATA: Drug Addiction Treatment Act
DEA: Drug Enforcement Administration
DSM-5: Diagnostic and Statistical Manual of Mental Disorders (Fifth Edition)
ED: emergency department
EHR: electronic health record
FQHC: Federally Qualified Health Centers
GPRA: Government Performance and Results Modernization Act
IRB: institutional review board
MAT: medication-assisted treatment
MOUD: medications for opioid use disorder
OUD: opioid use disorder
PDMP: prescription drug monitoring program
RRC: Recovery Resource Center
SAMHSA: Substance Abuse and Mental Health Services Administration
SUD: substance use disorder
UAB: University of Alabama at Birmingham
UDT: urine drug test
