# Supplementary material for: Development of an Emergency Department–Based Intervention to Expand Access to Medications for Opioid Use Disorder in a Medicaid Nonexpansion Setting: Protocol for Engagement and Community Collaboration
Source: JMIR Res Protoc. 2021 Apr 29;10(4):e18734. doi: 10.2196/18734 (PMC8120420; doi:10.2196/18734)
Supplement: Multimedia Appendix 1 [file resprot_v10i4e18734_app1.docx]

*Appendix****.*** *ED MOUD Induction Flowsheet*

COWS > 8 (Mod or Severe Withdrawal)

Contact research staff for assessment and linkage/peer navigator coordination in ED.

**Provide Buprenorphine/Naloxone induction in the ED:**

1)Give 4mg/1mg SL tab

2) Repeat in 60 minutes if patient still displays symptoms.

Contact research staff for assessment and linkage/peer navigator coordination in ED.

No

If female, is the patient’s urine pregnancy test positive?

Patient presents with opioid withdrawal symptoms, narcotic seeking, or an infectious complication of IV opioid abuse

COWS < 8 (No or Mild Withdrawal)

**Assess for withdrawal using COWS score.**

Yes

No

Yes

Does the patient have DSM-5 criteria for Opioid Use Disorder (OUD)?
